# Loss of Trust May Never Heal. Institutional Trust in Disaster Victims in a Long-Term Perspective: Associations With Social Support and Mental Health

**DOI:** 10.3389/fpsyg.2018.01204

**Published:** 2018-07-16

**Authors:** Siri Thoresen, Marianne S. Birkeland, Tore Wentzel-Larsen, Ines Blix

**Affiliations:** ^1^Norwegian Centre for Violence and Traumatic Stress Studies, Oslo, Norway; ^2^Center for Child and Adolescent Mental Health, Eastern and Southern Norway, Oslo, Norway

**Keywords:** institutional trust, disasters, mental health, social support, bereaved survivors, disaster survivors, psychotraumatic experience

## Abstract

Natural disasters, technological disasters, and terrorist attacks have an extensive aftermath, often involving society’s institutions such as the legal system and the police. Victims’ perceptions of institutional trustworthiness may impact their potential for healing. This cross-sectional study investigates institutional trust, health, and social support in victims of a disaster that occurred in 1990. We conducted face-to-face interviews with 184 survivors and bereaved, with a 60% response rate 26 years after the disaster. Levels of trust in the police and in the justice system were compared with general population data. We assessed the relationships between institutional trust and current psychological distress, social support, and life satisfaction. The levels of trust in the police and in the justice system were notably lower in survivors and bereaved than in the general population. Among the victims, low institutional trust was associated with more mental health problems, poorer social support, more barriers to seeking social support, and a lower life satisfaction. Lost trust in the aftermath of a disaster may perhaps never be restored and the lack of trust may act to strengthen or maintain health problems. An exclusively individualistic approach to trauma and disaster may miss out on the opportunities for promoting health and well-being that lies within the larger societal structures. Decision-makers should take this information into account, and acknowledge the potential long-term consequences of institutional performance in the aftermath of a disaster.

## Introduction

Most people will experience deeply distressing or traumatic experiences during their lifetime (Kessler et al., 1995; de Vries and Olff, 2009). These experiences stand apart from our ordinary daily life, and the way we react and cope with them can have the power to shape our future. It is well-established that aspects of the event itself, such as life threat; and personal resources, such as strong social bonds, affect how we cope in the face of adversity (Brewin et al., 2000; Ozer et al., 2008). This body of research has, however, been criticized for being overly individualistic and ignoring the wider social context in which the events are embedded (Ajdukovic, 2004). Natural disasters, technological disasters, or terrorist attacks have an extensive aftermath, often involving society’s institutions. How the police, justice system, health authorities, and other institutions handle the event may impact the victims’ potential for healing (Chandra et al., 2011). Not much is known, however, about the relationship between institutional trust and long-term coping following disasters. This study targets this knowledge gap by investigating institutional trust and its relation to well-being in survivors and bereaved from a ferry disaster that occurred in 1990.

Trust is a many-faceted phenomenon that can influence our behavior toward other people, organizations, and institutions. From a psychological perspective, basic trust is first acquired in interactions with reliable caregivers (Rotter, 1980). Thus, early life experiences lay the foundation for a belief that other people can be trusted. Generalized trust seems to be stable over time and is robust against personal experiences in adult life, with the exception of extreme situations (Uslaner, 2015). According to Uslaner (2015), trust in specific people (interpersonal trust) is more fragile than generalized trust, as it is based upon personal experience and depends on reciprocity. Similarly, institutional trust depends upon perceived trustworthiness and may change over time.

Institutional trust has been defined as the perceived probability that institutions will carry out their remit to a satisfactory degree (Hudson, 2006). Several other definitions of the term exist, many of which require a trustor (a subject, in our study an individual), a trustee (an object, in our study the police and the justice system), and some kind of expectation or evaluation, as in the formula “A trusts B to do X” (Hardin, 1993; PytlikZillig and Kimbrough, 2016). Both interpersonal trust and institutional trust seem to be related to happiness and well-being (Freitag, 2003; Hudson, 2006). However, the relationship between institutional trust and health is not clear and needs further investigation (Lindstrom and Mohseni, 2009; Giordano and Lindström, 2011).

When disaster strikes, the community mobilizes its institutional and organizational resources to deal with the immediate critical situation. Disasters occur in specific places and contexts. The pre-existing social, economic, and political fabric of the community may impact its resilience and adaptive ability (Cutter et al., 2008), and social or economic inequality can result in uneven recovery within communities (Rumbach et al., 2016). The ability of the particular community and the larger social system to provide the necessary resources in the post-disaster phase may also act to strengthen resilience over time (Norris et al., 2008).

Large-scale disasters and terrorist attacks may sometimes stimulate an increased sense of connectedness within the community, increased trust in authorities, and a “rally around the flag” mentality (Putnam, 2002; Dinesen and Jæger, 2013). Such increased trust is presumably short-lived (Wollebæk et al., 2012). In the long-term aftermath, institutions need to deal with the causes and consequences of the disaster. Issues such as blame, responsibility, compensation and aid to victims may invite distrust and conflict that can drag on for years, or even decades (Bos et al., 2005). Authorities may be perceived as non-responsive and evasive, and victims may become suspicious or may feel let down (Freudenburg, 1997). Governments may, perhaps unwillingly, need to allocate resources to prolonged, unresolved issues related to the disaster due to bottom–up pressure from victim groups (Bos et al., 2005). Victims may feel that the authorities have neglected their needs, that no one will accept responsibility, and that not much is being done to protect against future disasters. Thus, disasters may have a major and lasting impact on the social and economic fabric of the affected communities and on the relationship between victims and institutions (Bos et al., 2005).

Such processes may lead disaster victims to lose their belief in a just and fair society (Freudenburg, 1997) along with a generalized loss of trust in institutions. This probably implies a sense of increased vulnerability, such as the perception that when bad things happen, institutions cannot be trusted to provide the necessary resources or take action to ensure safety or justice. A perception of threat is thus carried forward in time, and may act to maintain psychological distress in the future (Ehlers and Clark, 2000). Previous research has documented that post-trauma health problems can continue for decades (Arnberg et al., 2011; Boe et al., 2011), and healing versus threat-maintaining environments may be key to individual health development. Embitterment may arise from a feeling of being treated unjustly, and the accompanying negative emotions such as anger or vengefulness (Nanni et al., 2018) may intensify other post-traumatic stress reactions.

Individuals who are victims of disaster may, as in the current study, have experienced a life-threatening event or have lost someone close. Thus, trust or lack of trust in the aftermath is connected to processes of high personal relevance, related to survival. Under these circumstances, a potential trust erosion will presumably be of high emotional value, leaving the individual feeling vulnerable, no longer believing that B will do X when A needs it most. In high-trust societies in particular, people in a crisis situation may feel strongly let down by institutions who, unexpectedly, did not come to their aid. In an interpersonal context, the feeling of being let down has been found to be a strong predictor of later mental health problems (Brewin and Holmes, 2003), and may also be relevant for our relationship with society and its institutions. Lost trust may take a long time to rebuild, and trust harmed by deception (or perceived deception) may never fully recover (Schweitzer et al., 2006).

Unexpected and extreme events of life threat and loss introduce the idea that our peaceful reality can become a nightmare in an instant, bringing chaos, horror, and loss of trust in the world as we know it. In such times of despair, our need for stability, structure, and support may be greater than what institutions are prepared for or can provide. Thus, psychological responses to the disaster may in themselves lead to more negative perceptions of support from authorities (Barnes et al., 2013). Victims may feel the need to regain control and make sense of the disastrous event, and, perhaps, to hold someone responsible (Whitson and Galinsky, 2008; Radnitz and Underwood, 2017), although issues of responsibility may be blurred and what actually happened may remain unclear. Some authors have argued that the post-disaster meaning-making process may result in suspiciousness (Sullivan et al., 2010), although it is not clear whether this is a product of institutional untrustworthiness. Thus, conflicts of interest or conflicting perceptions of reality may easily arise between victims and institutions. Perceived institutional wrongdoings, ignorance, or lack of interest may leave a lasting emotional footprint in victims, with potential consequences for their future health development.

Erosion of institutional trust following trauma may relate not only to victims’ health, but also to the quality of social bonds and social support. If institutional trust is harmed in trauma victims, but not in society at large, victims may feel estranged from their communities or feel an “experiential dissimilarity” that sets them apart from others (Thoits, 2011). Previous research has pinpointed how social connectedness can be threatened in the aftermath of trauma (Arnberg et al., 2013). Loss of institutional trust may add to interpersonal difficulties by inhibiting support-seeking, particularly over time, because the individual may feel that others are tired of hearing about it, or that they cannot understand them. Such processes have been found to link closely to poor mental health (Thoresen et al., 2014).

Accordingly, we have reason to believe that institutional trust may be shattered in disaster victims, with potential negative implications for health and well-being over time. Research in this area is lacking, particularly relating to long-term adjustment. We intend to address this knowledge gap by investigating institutional trust and health in victims of a ferry disaster that occurred in 1990.

The purpose of this study was to investigate the relevance of institutional trust for long-term adjustment after trauma. We used data gathered in a face-to-face interview study with survivors and bereaved from the with Scandinavian Star ferry disaster to address the following aims: (1) Estimate the potential difference in level of institutional trust between survivors and the general population. (2) Assess potential links between disaster-related trust in authorities and current institutional trust. (3) Determine the potential relationships between institutional trust and current social support, psychological distress, and life satisfaction.

## Materials and Methods

### The Scandinavian Star Ferry Disaster and Its Aftermath

During the night of April 7 1990 a fire broke out on the passenger ferry Scandinavian Star. This disaster resulted in the death of 159 people and an unborn child people (one out of three passengers died). The majority of the passengers were families on easter vacation and sport clubs on their way to training camps. Hence, this disaster resulted in the deaths of many young people. Although the police concluded that the fire was most likely arson, the perpetrator(s) was never identified, and the aftermath of the disaster has been riddled with conflict. Some of the issues that have been fiercely debated include questions regarding ferry ownership and safety responsibilities, the poor state of the ship, the lack of disaster preparedness on board, the governmental responsibility for safety at sea, the quality of the police investigations, victim compensation, and health assistance to victims. To date, the fire remains an unsolved crime, and has received regular media attention for almost three decades. This disaster did not entail loss of physical resources such as destroyed housing or infrastructure. The event occurred in a high-income and high-equality country with an accessible and affordable public health system. Following requests from survivor groups, the Norwegian Parliament appointed an independent commission in 2015 with the mandate to evaluate several aspects of the Scandinavian Star case. As part of their work, in 2016 the commission requested a systematic investigation of the current mental health status of survivors and bereaved from the ferry disaster.

### Participants and Procedures

The commission supplied lists of with Norwegian survivors who were on board the ship at the time of the fire and still alive in 2016 (*n* = 168). The bereaved group consisted of individuals who received compensation settlements from the ship owner’s insurance company (*n* = 205), next-of-kin who did not receive compensation but were identified by the national support group (*n* = 49), and next-of-kin who self-recruited to the study (*n* = 4). Among these 258 bereaved individuals, nine had been on board the ship and were included in the list of survivors, resulting in a total of 249 bereaved who were not on board the ship, of which 186 were still alive. Of the 168 survivors and the 186 bereaved (354 in total), we were not able to find contact information in 22 cases, and an additional 11 persons were not capable of participating due to illness, leaving 321 individuals eligible for the study. Of these 321, 193 participated (98 survivors and 95 bereaved), resulting in a response rate of 60% (in total, and for survivors and bereaved alike).

We did not have much information for comparing responders and non-responders in this study. Of the 321 individuals we tried to contact, more women (98 out of 145 = 68%) than men (95 out of 176 = 54%) participated. Among survivors, 23% reported having been located in the deadliest parts of the ship (pre-defined according to certain corridors on certain decks) at the time the fire started, compared to the original police investigation placing 28% in these areas. Among the bereaved study participants, 70% had lost a partner, child, parent, and/or a sibling in the fire, compared to 62% among the bereaved who did not participate in the study.

Survivors and bereaved were mailed invitations to participate in the study. Those who did not opt out of the study were subsequently contacted by phone. Face-to-face interviews were conducted in our offices or in the participants’ home in the period October–December 2016. Interviewers were health personnel; the majority had previous experience with conducting research interviews, and all had participated in a 1-day training seminar for this study. All information was recorded on a tablet and was transferred encrypted without local storage to the University of Oslo server for sensitive data. The interviewee gave written consent to participate in the report to the Norwegian Parliament. Most participants (96%, *N* = 185, 94 survivors and 91 bereaved) gave their additional written consent to use the information they had provided for research purposes. The Regional Committee for Medical and Health Research Ethics approved the study, and our procedures included a follow-up strategy for participants in distress.

### Comparison Material: The European Social Survey (ESS)

We compared the level of institutional trust in survivors and bereaved with general population data from the European Social Survey (ESS). The ESS is a cross-national survey that was established in 2001. Every 2 years, face-to-face interviews are conducted in cross-sectional samples in more than thirty European countries, allowing for comparisons of attitudes, beliefs, and behavior across nations and over time. The survey specifications aim to unify methods of inquiry, sampling, data collection and data processing, and samples are presumably representative of citizens above the age of 15. In Norway, the data is gathered by statistics, Norway. ESS data are freely accessible on their home page, and more information about survey methods is available on the ESS home page^1^. Data round seven, gathered in 2014 and made available in 2015, was the most recent data set at the time of this study. More information about the Norwegian surveys can be downloaded from http://www.europeansocialsurvey.org/data/download.html?r=7.

### Measures

#### Institutional Trust

Trust in police and trust in the justice system were measured by two items from the ESS: (1) “How much do you personally trust the police?” and (2) “How much do you personally trust the legal system?” Both items are scored on a scale from 0 (no trust at all) to 10 (complete trust). The current study applied the question formulation used in the ESS.

Disaster-specific trust was measured by several items tapping into the perceived performance of authorities and society in relation to how they handled the disaster aftermath: (1) trust in the first police investigation (1990–1991); (2) trust in the second police investigation (2014–2016); (3) perceived capability of the health authorities to make sufficient follow-up available to the disaster victims; (4) perceived ability of the Norwegian society to supply the victims with the support they needed; and (5) perceived interest from the Norwegian authorities in the victims’ experiences and opinions. We could not ask questions about disaster-specific trust in the justice system, as the arson case never came to trial. All items were scored on a scale from 1 (not at all) to 5 (very much).

Traumatic exposure in survivors was measured by several dichotomous (yes/no) single items constructed for this study, including whether or not the survivor had lost someone they knew to the fire, had been present in areas with heavy smoke, saw someone injured or dead, heard people screaming for help, or experienced additional dangerous situations during the evacuation of the ship.

Pre-disaster exposure to interpersonal violence was measured by 6 adapted dichotomous (yes/no) items from the Stressful Life Event Screening Questionnaire (SLESQ, Goodman et al., 1998): Forced sexual acts; other forms of sexual assault; childhood physical violence (kicked, beaten up, or otherwise attacked or harmed by parental figures or other grown-ups); adult physical violence (kicked, beaten up, or otherwise physically harmed); psychological violence from family member or partner (repeatedly ridiculed, humiliated, or told you’re no good); and witnessing (witnessing another person being seriously injured, killed, maltreated, or sexually assaulted). For the purpose of this study, we computed a dichotomous variable in which any severe pre-disaster violent interpersonal event (1) was contrasted with no such event (0).

Perceived social support was measured by the Crisis Support Scale (Joseph et al., 1992) and included the following four questions with a response format on a five-point scale ranging from 1 (never) to 5 (very often/always): (1) When you feel the need to talk, how often is someone willing to listen to you?, (2) Are you able to talk about your thoughts and feelings?, (3) Do people show you sympathy and support?, and (4) Is there someone who can give you practical help? Cronbach’s alpha was 0.80.

#### Social Support Barriers

Respondents were asked to what degree they had refrained from seeking help or support or from talking about their situation with other people because they thought (1) that people were tired of hearing about it, (2) that other people had enough dealing with their own problems, (3) that people would think they were too caught up with it, (4) that they would be burdening their friends, or (5) that people wouldn’t understand them (Thoresen et al., 2014). All items were scored on a five-point scale from 1 (not at all) to 5 (very much). Cronbach’s alpha was 0.91 in the current sample.

#### Post-traumatic Stress Reactions in the Last Month

The Post-traumatic Check List (PCL-5) is a 20-item self-administered questionnaire that assesses DSM-5 PTSD symptoms (Blevins et al., 2015). We used the PCL-S (specific), and the items were specifically linked to the fire on Scandinavian Star. The participants were asked to indicate the extent to which they had been bothered by each symptom during the last month, on a scale ranging from 0 (not at all) to 4 (extremely). Cronbach’s alpha for the total scale was 0.94.

Psychological distress in the last week was measured by a 10-item version of the Hopkins Symptom Checklist (HSCL; Derogatis et al., 1974). Five items measure symptoms of depression: (1) Feeling hopeless about the future, (2) Feeling blue, (3) Blaming yourself for things, (4) Feeling everything is an effort, and (5) Feelings of worthlessness. Another five items measure anxiety: (1) Suddenly scared for no reason, (2) Faintness, dizziness or weakness, (3) Feeling fearful, (4) Feeling tense or keyed up, and (5) Difficulties falling asleep or staying asleep. Responses are recorded on a scale from 1 (not bothered) to 4 (bothered a great deal). This abbreviated version of the HSCL has shown good psychometric properties, and has previously been found to correlate highly (*r* = 0.97) with the HSCL-25 in a general population sample (Tambs and Moum, 1993). Cronbach’s alpha for this 10-item scale was 0.93.

#### Life Satisfaction

We used Diener’s Satisfaction with Life Scale (SWLS) (Diener et al., 1985) which includes five statements: (1) In most ways my life is close to my ideal, (2) The conditions of my life are excellent, (3) I am satisfied with my life, (4) So far I have gotten the important things I want in life, and (5) If I could live my life over, I would change almost nothing. Participants indicate the degree to which each statement applies to them, ranging from 1 (strongly disagree) to 7 (strongly agree). For the purpose of the current paper, we used a mean score of the five items, with a Cronbach’s alpha of 0.93.

Demographic information included gender, age, marital status, and perceived financial situation. The sample had no ethnic diversity.

### Statistical Analysis

#### Comparisons With the European Social Survey (ESS)

Of the 185 participants who consented to having the data used for research purposes, one individual had missing information on age, resulting in 184 participants eligible for comparison with the ESS survey data. The ESS national data for 2014 included 1156 individuals in the age range represented in the Scandinavian Star study (27–89 years of age). Of these 1156, four individuals had missing information on trust in police and/or trust in the legal system, resulting in a comparison material of 1153 individuals.

In the comparison of institutional trust in our sample with the general population sample (ESS data), we present the results unweighted, with design weights only, and with design- and post-stratification weights, as recommended by ESS. The purpose of design weights is to correct for a possible sample selection bias. The design weights are computed as the inverse of the inclusion probabilities and then scaled in such a way that their sum equals the net sample size. Post-stratification weights uses auxiliary information to reduce the sampling error and potential non-response bias, and have been constructed on the basis of information on age-group, gender, education, and region. Post-stratification weights can reduce the sampling error and a potential non-response bias. More information about the ESS weighting can be found on their home page^2^. The design- and post-stratification weights recommended by ESS were divided by their means in the comparison material so that their means were still 1. In the disaster sample, all weights were set to 1. Differences between levels of institutional trust in disaster victims and in the ESS general population sample were tested with linear regression models with 95% confidence intervals. We present unadjusted and gender- and age-adjusted, as well as weighted and unweighted, comparisons of trust levels.

#### Correlations

We used Pearson correlations to estimate associations between variables in the study. Linear regressions were used to investigate associations between institutional trust and current psychosocial adjustment, adjusting for age, gender, and pre-disaster violence exposure.

Correlations and linear regressions were performed in IBM SPSS statistics for Windows, version 20. Comparisons with ESS were performed in R (The R Foundation for Statistical Computing, Vienna, Austria, 2013).

## Results

The 184 participants comprised 94 women (51.1%) and 90 men (48.9%). Their mean age at the time of the interview was 55 years (range: 27 to 89). Thus, their mean age at the time of the disaster in 1990 was 29, ranging from small children to older adults. At the time of the study, the majority of participants were married or cohabitating with a romantic partner (68.5%, *n* = 126), had 12 or more years of education (64.7%, *n* = 119), and perceived their financial status to be average or above average (89.5%, *n* = 162).

About half the sample (50.5%, *n* = 93) were on board the ferry at the time of the fire, while the others (49.5%, *n* = 91) had not been on the boat, but had lost one or more family members to the fire. The traumatic exposure in the 93 survivors was high, as 33.3% (31) knew someone that died in the fire, 76.3% (*n* = 71) were in areas of the boat with heavy smoke, 41.9% (*n* = 39) heard people screaming or calling for help, 35.5% (*n* = 33) saw somebody wounded or dead, 62.4% (*n* = 58) experienced a dangerous situation during the evacuation of the ship, and 19.4% (*n* = 18) thought they were going to die.

### Erosion of Institutional Trust? Comparison With a General Population Study

In the Scandinavian Star sample, the mean level of trust in the police and in the justice system was 5.3 (*SD* = 2.8) and 5.6 (*SD* = 2.9), respectively, both on a scale ranging from 0 to 10. In the general population sample (unweighted and unadjusted), the corresponding numbers were 7.4 (*SD* = 2.0) and 7.2 (*SD* = 2.0). **Table 1** displays the results of a series of linear regression models with unweighted and weighted population data, with and without adjustment for age and gender. Trust in the police in the Scandinavian Star sample was approximately 2 points lower, and the trust in the justice system approximately 1.4 points lower than that of the general population sample. The size of the difference between the Scandinavian Star sample and the general population equalled approximately one standard deviation in the general population sample for trust in the police, and somewhat less than a standard deviation for trust in the justice system. There were no significant differences in levels of trust between survivors (trust in police: mean = 5.4, *SD* = 2.9, trust in the justice system: mean = 5.9, *SD* = 2.8) and bereaved (trust in police: mean = 5.1, *SD* = 2.8, trust in the justice system: mean = 5.4, *SD* = 3.1) (*t*-test *p*-values 0.444 and 0.321).

**Table 1:** Differences in institutional trust between the (Scandinavian Star) sample and the general population data, in weighted and unweighted population data and in unadjusted and age and gender-adjusted models.

Unweighted and weighted models for trust in the police and in the justice system	Unadjusted		Adjusted for age and gender	
	Regression coefficient	95% CI	Regression coefficient	95% CI
**Trust in the police (range 0–10)**				
Unweighted	-2.02	-2.34, -1.69	-2.03	-2.36, -1.70
Design weights only	-2.02	-2.34, -1.69	-2.03	-2.36, -1.70
Design and post-stratification weights	-2.00	-2.33, -1.67	-2.02	-2.34, -1.69
**Trust in the justice system (range 0–10)**				
Unweighted	-1.53	-1.87, -1.19	-1.50	-1.84, -1.16
Design weights only	-1.53	-1.87, -1.19	-1.50	-1.84, -1.16
Design and post-stratification weights	-1.45	-1.79, -1.12	-1.43	-1.77, -1.09

*All p-values < 0.001.*

### Trust in How the Authorities Handled the Disaster Aftermath, and Its Relation to Institutional Trust

Participants expressed predominantly negative perceptions of how the authorities handled the aftermath of the disaster (**Table 2**). The majority reported a lack of trust in the two police investigations, particularly the first. The support from society and health authorities was only highly regarded by a small minority.

**Table 2:** Participants’ perceptions of how the authorities handled the disaster aftermath.

Aspects	Negative (not at all/low)		Neutral (partly)		Positive (high/very high)	
	%	*n*	%	*n*	%	*n*
Trust in the first police investigation (1990–1991)	77.7	143	15.2	28	7.1	13
Trust in the second police investigation (2014–2016)	59.8	110	28.3	52	12.0	22
Health authorities made sufficient follow-up available to victims	61.9	112	28.2	51	9.9	18
The victims have received the support they needed from society	53.3	96	39.4	71	7.2	13
The Norwegian authorities have shown an interest in the victims’ experiences and opinions	58.2	106	31.3	57	10.4	19

In **Table 3**, we examine the associations between disaster-specific perceptions of the authorities and current institutional trust in the police and the justice system. Trust in the police was highly correlated with trust in the justice system. Trust in the police and the justice system were both significantly and positively, although moderately, associated with each of the disaster aftermath-related items.

**Table 3:** Correlations between perceptions about how authorities handled the disaster aftermath and current institutional trust.

	1	2	3	4	5	6
(1) Trust in the police	–					
(2) Trust in the justice system	0.84					
(3) Trust in the first police investigation (1990–1991)	0.51	0.47				
(4) Trust in the second police investigation (2014–2016)	0.48	0.43	0.62			
(5) Health authorities made sufficient follow-up available to victims	0.22	0.26	0.22	0.30		
(6) The victims have received the support they needed from society	0.26	0.29	0.26	0.30	0.66	
(7) The Norwegian authorities have shown an interest in the victims’ experiences and opinions	0.28	0.29	0.29	0.35	0.56	0.51

*All p-values ranged from <0.001 to 0.003.*

### Institutional Trust and Psychosocial Adjustment

Trust in the police and trust in the justice system were significantly associated with current psychosocial adjustment (**Tables 4**, **5**). The associations were low for social support and psychological distress, but of moderate magnitude for social support barriers, post-traumatic stress, and life satisfaction.

**Table 4:** Associations between institutional trust and social support.

Institutional trust	Perceived social support				Social support barriers			
	*B*	95% CI	*p*-Value	β	*B*	95% CI	*p*-Value	β
Trust in the police	0.05	0.001, 0.09	0.017	0.18	-0.12	-0.17, -0.07	<0.001	-0.34
Trust in the justice system	0.06	0.02, 0.10	0.006	0.20	-0.12	-0.17, -0.07	<0.001	-0.35

*Linear regressions. Adjusted for age, gender, and pre-disaster interpersonal violence; B, unstandardized regression coefficient; β, standardized regression coefficient.*

**Table 5:** Associations between institutional trust and well-being.

Institutional trust	Post-traumatic stress				Psychological distress				Life satisfaction			
	*B*	95% CI	*p*-Value	β	*B*	95% CI	*p*-Value	β	*B*	95% CI	*p*-Value	β
Trust in the police	-0.11	-0.15, -0.07	<0.001	-0.40	-0.06	-0.09, -0.03	<0.001	-0.28	0.14	0.07, 0.21	<0.001	0.27
Trust in the justice system	-0.11	-0.15, -0.08	<0.001	-0.43	-0.06	-0.09, -0.03	<0.001	-0.26	0.15	0.08, 0.22	<0.001	0.30

*Linear regressions. Adjusted for age, gender, and pre-disaster interpersonal violence; B, unstandardized regression coefficient; β, standardized regression coefficient.*

## Discussion

This is the first study to investigate institutional trust in disaster victims in a very long-term perspective. The levels of institutional trust were notably lower in victims compared to the general population almost three decades after a ferry disaster. This was observed for trust in the police as well as for trust in the justice system. It has previously been noted that those personally affected by a disaster show particularly low trust in government (Hommerich, 2012). Chronic negative consequences may arise from poor communication between evasive and non-responsive authorities and suspicious and disappointed victims (Arata et al., 2000). For example, following the Sewol Ferry disaster, no improvement in reactive embitterment was identified over a 3-year period (Chae et al., 2018). Individuals who have personal experiences with (perceived) untrustworthy authorities in situations of strong negative emotional valence may never regain their trust. Institutional trust is thought to be an important asset in coping with disaster (Hommerich, 2012), and loss of trust may represent a significant loss of resources (Hobfoll, 2001), thereby harming people’s ability to recover.

Trust in the police and the justice systems in Norway are among the highest in a European context (Kleven, 2016). Trust is usually conceived of as something good, as a strength or an asset, but some scholars have pointed to what they call the dark side of trust (Neal et al., 2016). The authors argue that if a trusted institution behaves in an untrustworthy way, deception, exploitation, or unfulfilled expectations may follow. In that sense, high-trusters have more to lose. These arguments are similar to Janoff-Bulman’s theory of shattered assumptions (Janoff-Bulman and Frieze, 1983), predicting that the larger the loss, the worse the outcome. However, it is not at all clear if it is the loss of trust or the lack of trust which is of importance. In low-trust societies, perceived institutional untrustworthiness may not represent any loss, but rather confirm and actualize negative expectations. Thus, disaster victims in both low-trust and high-trust societies may lack institutional trust, and this lack may have negative consequences irrespective of the level of pre-disaster trust. In high-trust societies, however, disaster victims who acquire a trust disruption may perceive themselves to be different from other people in their social network, and they might find it difficult to feel understood and acknowledged. Whether, it is the loss of trust or the lack of trust that is most relevant for people’s health and well-being remains to be investigated.

Previous scholars have pointed to the importance of place for understanding resilience to disasters (Cutter et al., 2008; Rumbach et al., 2016). In the current study, the disaster took place within a high-income and high-equality society. Community and societal factors may have very complicated relationships to post-traumatic distress. For example, although relative social disadvantage is related to an increased illness risk (Koenen et al., 2017), low income countries seem to actually have a lower conditional risk of mental health problems following trauma (Dückers et al., 2016). In contrast to several other types of collective disasters such as hurricanes, floods, or tsunamis, this disaster did not entail loss of physical resources such as destroyed housing or infrastructure. The disaster victims returned to their intact local communities and neighborhoods, but also to a social network that did not share their fate. In this study, we could not address the importance of these factors, but future cross-national studies could perhaps disentangle the relationships between trust and well-being across events, places, and populations.

In our study, the disaster victims displayed overall negative opinions regarding the authorities’ handling of the disaster, including their perceptions of the police investigations, the health authorities, and the society at large. Considering that the data was collected 26 years after the disaster, it is important to note that these perceptions could reflect current opinions rather than previous ones. These negative perceptions were significantly associated with institutional trust, indicating that the particular disaster-related experiences may have affected general trust, keeping in mind that causal directions could not be determined in this study. When changes occur in institutional trust, they may generalize across institutions (Dinesen and Jæger, 2013). In our study, correlations with opinions about how the authorities handled the disaster were highly similar for trust in the police and trust in the justice system, consistent with the generalization hypothesis.

Almost three decades after the disaster, low institutional trust was associated with more mental health problems, poorer social support, more barriers to seeking social support, and a lower life satisfaction. These results lend support to previous observations that victim stress may result in part from negative experiences with authorities in the post-disaster aftermath (Freudenburg, 1997; Arata et al., 2000; Bos et al., 2005; Chae et al., 2018). If disaster victims learn that it is futile to turn to institutions for help in matters of high emotional value, an accompanying sense of being unprotected may act to maintain mental health problems over time. Victims may also feel a need to communicate their experiences within their social network and express their worries about institutional unresponsiveness. In this process, they run the risk of rejection or a lack of understanding from people who do not share their experiences and perceive the institutions differently. As a result the victims may feel alienated and limit their social support-seeking (Arnberg et al., 2013; Thoresen et al., 2014), weakening the social bonds that are so important for post-disaster health.

### Strengths and Limitations

Due to the cross-sectional design, no causal inferences could be drawn, and longitudinal studies are needed to explore temporal relationships between the study factors. The cross-sectional data also restricted our ability to adjust for confounders, as we would risk over-adjustment by including factors that may have a role in a potential causal chain. Considering the number of years that have passed since the disaster, we deem the response rate of 60% to be good, however, we had little information about non-responders. Women were somewhat over-represented among responders, but the sample seemed to be fairly representative in relation to the exposure level (survivors) and relations to the deceased (bereaved). We compared the level of trust in survivors and bereaved who were interviewed in 2016 with general population data from 2014. However, trust in police and the justice system have remained remarkably stable in Norway during the 12-year period it has been measured by ESS.

Scholars have argued that “have-nots” have lower institutional trust than “haves” (Hudson, 2006). However, we do not believe that the observed differences between our sample and the general population can be attributed to demographic variables, as we did not find indications that the survivors and bereaved from the Scandinavian Star disaster were a particularly poor, unemployed, or otherwise marginalized group.

The context of this study was the public inquiry into several aspects of the Scandinavian Star case, and the results should be interpreted within this context. The interview constituted an opportunity for survivors and bereaved to voice their worries or dissatisfaction. The public inquiry was initiated in response to harsh criticism of how the authorities, in particular the police, handled the disaster, and confirmation bias might have played a role in this particular context. Participation in this study involved many questions about what happened during and after the fire, and it may be that recalling these difficult events influenced the respondents’ emotional states and their answers regarding current trust in authorities. Furthermore, consistency bias might have played a role in the present study, as many participants expressed their dissatisfaction with the help they received in the aftermath of the disaster.

The focus of this study was on victims’ perception of their situation, and all data was self-reported. It was not within the scope of our study to evaluate the institutional performance in the Scandinavian Star case. However it should be noted that the final report from the governmental-appointed independent commission (published after the data collection in the present study) was somewhat critical of the authorities’ support of the disaster victims.

The current study focused on a specific disaster. Further, this particular study concerned victims of a disaster which has been followed by conflict and unresolved issues. In disasters followed by a low level of conflict, institutional trust may not be a relevant issue for victims’ well-being. Our participants had experienced loss and trauma, which are common features of many disasters. Nevertheless, they returned to an intact neighborhood, whereas many other disasters result in great material destruction. Thus, future studies are needed to investigate the generalizability of our results. It would also be valuable to investigate if similar results can be found in victims of other types of trauma, such as rape and physical assault.

The Scandinavian countries have small and stable populations, making it possible to reach people many years after an exposure. Other strengths of this study included the high response rate, the sufficient sample size, the comparison with general population data, and a remarkably low level of missing data. This is also the first study to investigate institutional trust in disaster victims in a long-term perspective.

## Conclusion

Almost three decades after a disaster, institutional trust was notably lower in disaster victims compared to the general population, and victims’ institutional trust was related to mental health, social support, and life satisfaction. The way institutions communicate with and reach out to disaster victims may strengthen or weaken their institutional trust. Continued trust may contribute to healing while a lack of trust can act to intensify or maintain health problems. Institutional trust, then, can be viewed as one of several important factors that may influence healing processes following disasters.

An individualistic approach to trauma and disaster may miss out on the opportunities for promoting health and well-being that lies within the larger societal structures. Health professionals may need to make their knowledge of how people cope with trauma available to decision-makers. Decision-makers should take this knowledge into account, and realize that institutional performance may influence victims’ well-being. From a clinical perspective, it may be beneficial to be aware of the potential impact of lost institutional trust in trauma victims, when it comes to health, well-being, and social relations.

## Ethics Statement

The study was approved by the Regional Committee for Medical and Health Research Ethics (REK) in Norway with the following reference number in the REK: 2016/1527/REK nord/REK North. Survivors and bereaved were mailed an information letter describing the study. Those who did not opt out of the study were subsequently contacted by phone and invited to take part in a face-to-face interview. Written informed consent was obtained for all participants. The study included a follow-up service for participants who might have felt distressed after completing the interview. To ensure confidentiality, data was registered on a tablet without local storage and transferred encrypted to the University of Oslo’s server for sensitive data (TSD). The research team has collaborated with the disaster support groups in designing the interview guide and promoting the study on National television. We have presented the study results for the support groups.

## Author Contributions

ST was the PI of the study and first draft of the paper. ST, MB, and IB designed the study and conducted the data collection. ST, MB, IB, and TW-L decided on the research questions, analytic strategy of the current manuscript, made substantial contributions to interpretation of the data, and revising the manuscript. ST and TW-L (MSc/statistician) conducted the analyses. All authors had full access to the data and have agreed to be accountable for all aspects of the work and have read and approved the final version of the manuscript.

## Conflict of Interest Statement

The authors declare that the research was conducted in the absence of any commercial or financial relationships that could be construed as a potential conflict of interest.

## Footnotes

1 http://www.europeansocialsurvey.org/about/

2 http://www.europeansocialsurvey.org/methodology/ess_methodology/data_processing_archiving/weighting.html
